# Integrated care by community health workers in Malawi: Rehabilitation and blood pressure monitoring

**DOI:** 10.3934/publichealth.2021009

**Published:** 2021-01-28

**Authors:** Athenie Galvez, Jordan Waite, Kyle Jureidini, Kathryn C Nesbit

**Affiliations:** 1 Presidio Sport & Medicine, San Francisco, CA, USA; 2 Orthopedic and Neurological Rehabilitation, Los Gatos, CA, USA; 3 Body Mechanix Physical Therapy, Simi Valley, CA, USA; 4 UCSF/SFSU Graduate Program in Physical Therapy, San Francisco, CA, USA

**Keywords:** community health workers, integrated care, palliative care, noncommunicable disease

## Abstract

**Background:**

Community health workers (CHWs) are essential providers of integrated care for people in low-resourced settings with a high burden from noncommunicable diseases (NCDs).

**Aims:**

The purpose of this study was to evaluate a CHW training program in rural Malawi integrating blood pressure (BP) monitoring into rehabilitation care.

**Methods:**

This was a retrospective cross-sectional study. The participants were a convenience sample of home-based palliative care CHWs at the local hospital (*n* = 59). Data collected included: a written pre- and post-knowledge test, skills competency checklist and a post-training program survey. Descriptive frequencies and paired *t*-tests (*a* = 0.05) were used for quantitative analyses. Themes in narrative responses in the post-training survey were analyzed qualitatively.

**Results:**

Participant knowledge regarding BP monitoring procedures improved significantly on the post-test (M = 8.24, SD = 1.654) compared to the pre-test (M = 6.59, SD = 1.683), Z (49) = −5.569, p < 0.001. The pre-and post-tests were scored 0–10 points. All participants demonstrated competency in 100% of the skills. Participants reported the lack of transportation, teamwork and resources as barriers to their work. They reported trainings and opportunities to collaborate as facilitators to their work.

**Discussion:**

This study demonstrated the effectiveness of a training program for CHWs which integrated BP monitoring with rehabilitation care for people with NCDs. This retention of knowledge and application to clinical practice serve as strong indicators of the feasibility and sustainability of the CHW training and care delivery program in resource-limited settings.

**Conclusion:**

Our findings help demonstrate that training CHWs can be an effective way to help bridge the gap in health care access for people with disabilities in resource-limited countries.

## 1. Introduction

The health burden of noncommunicable diseases and injuries (NCDIs) is growing even as healthcare in low-income countries remains focused on communicable, maternal, neonatal, and nutritional conditions. In fact, NCDIs contributed to 79.8% of overall global mortality in 2015, with over half of those deaths occurring in low- and middle-income countries [1]. To reduce inequities in care, health systems need to intentionally prioritize the needs of people with noncommunicable diseases [2],[3]. Health workers who provide care for those with NCDIs are in short supply [4]. However, community health workers (CHWs) play an integral role in filling this gap. CHWs are health care generalists who serve the communities in which they live [5]. They are an effective and low-cost subsystem of health workers in sub-Saharan Africa [6]. Malawi, a low-income country in southeast Africa, has a population of 21.2 million with 75% of the country's population living below the poverty line [7],[8]. Four percent of the population live with a physical disability and 22% of these persons have difficulty walking [6]. Malawi has a heavy burden of disease. In 2015, estimates suggest that Malawi lost 58,000 disability adjusted life years (DALYs) per 100,000 population when accounting for all diseases [1]. NCDIs account for approximately one third of all deaths and disabilities in Malawi [1]. Therefore, consideration of NCDI risk factors, interventions, and prevention policies is critical [1],[9].

Located in Namitete, Malawi, a rural hospital serves over 250,000 people within a 60km radius [10]. This is one of the few hospitals in the country that offers a home-based palliative care (HBPC) program. This program serves patients in the village with a variety of life-limiting or life-threatening conditions and includes a HBPC coordinator and 60 volunteer CHWs with specialized training in palliative care. Trainings were held annually since 2013 with a focus on rehabilitation skills so that the CHW could better serve patients who were bed-ridden or had physical disabilities. In recent years, the hospital staff noted an increase in patients with stroke or second stroke and wanted to initiate strategies to mitigate this trend. High blood pressure (BP) was identified as an indicator of risk of stroke or second stroke. The stakeholders at the hospital (medical director and HBPC coordinator) wanted the CHW trainings to include BP monitoring because many of the patients in the HBPC program have NCDs with related BP issues. In addition, the CHWs in the HBPC program reach the doorsteps of these patients providing access to care in the large catchment area. They saw the opportunity for the CHWs to increase appropriate referrals to the hospital and monitor BP issues in conjunction with their rehabilitative care in the village.

For the training evaluated in this study, the training content was unique to this program. It was developed in collaboration with the hospital staff, HBPC coordinator, a licensed physical therapist, student physical therapists and CHWs. All written training materials and assessments were translated into Chichewa by native Chichewa speakers. The training was delivered with Chichewa interpreters as needed. The purpose of this study was to evaluate a CHW training program that integrated BP monitoring into their rehabilitation care in the home-based setting.

## 2. Methods

### 2.1. Study Design

This is a retrospective, cross-sectional study of a training program in December 2019.

### 2.2. Subjects

The participants were a convenience sample of all active home-based palliative care community health workers at a local hospital in Namitete, Malawi (n = 61). Table 1 is a summary of their known demographic characteristics.

**Table 1.** Participant demographic characteristics.

	Group 1 n/N (%)	Group 2 n/N (%)	All n/N (%)
Gender			
Female	19/28 (68%)	16/31 (52%)	25/59 (42%)
Male	9/28 (32%)	15/31 (48%)	34/59 (58%)
Mean Age (SD)	47.89 (7.661)	54.94 (9.842)	51.59 (9.487)
Region			
TA Kalolo	18/28 (64%)	21/31 (68%)	39/59 (66%)
TA Mavwere	10/28 (36%)	10/31 (32%)	20/59 (34%)

Note: TA = Traditional Authority.

### 2.3. Training planning

The training content was developed in collaboration with the medical director, HBPC coordinator and 5 CHWs in December 2018. To evaluate the feasibility of CHW BP monitoring, 5 BP machines were distributed to 5 CHWs in 5 different geographic areas. The CHWs were assessed for correct BP monitoring (30 observations). Of 30 observations, all CHWs measured BP correctly without physical assistance (one received verbal directions), all CHWs except one accurately entered the BP information in the patient's health book (one observation with no response). This training planning established the feasibility of training CHWs in BP monitoring and informed the planning for the full training in December 2019. Based on the input from the CHWs involved in the planning, instructions were clarified, documentation processes developed, and assessment measures determined.

### 2.4. Training program details

**Figure 1.**
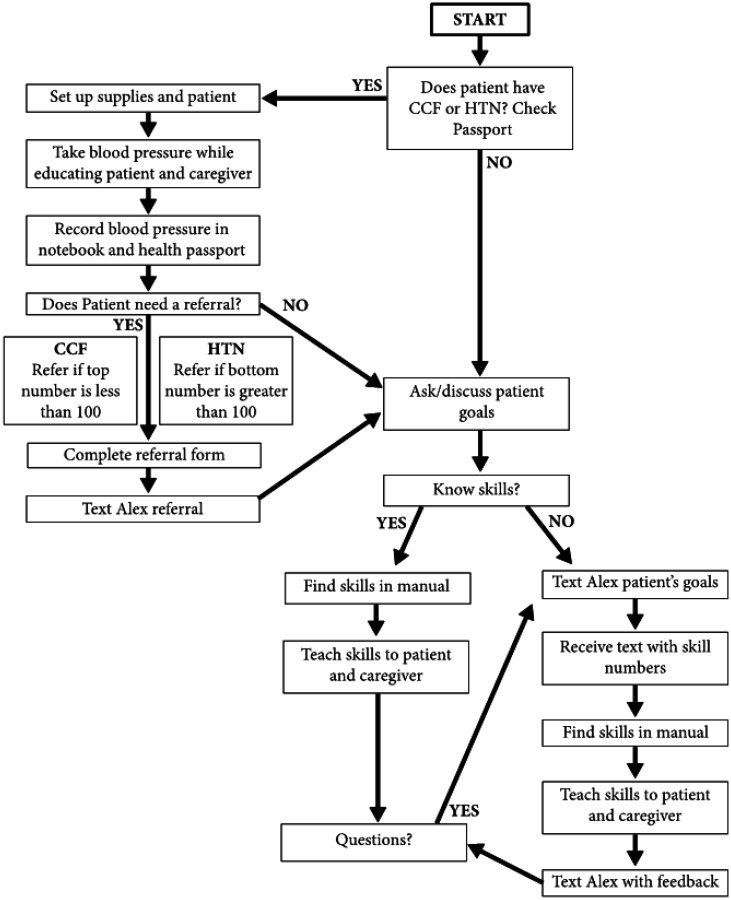
Integrated Care Workflow. Note: CCF = Chronic Cardiac Failure, HTN = Hypertension, Alex = Home-Based Palliative Care coordinator.

The training program included 2 days of instruction on NCDs, BP monitoring, criteria for referral to the hospital, documentation of BP monitoring, as well as stroke prevention education and rehabilitation skill review. The type of content included knowledge, psychomotor skill, application to work processes and cases. The teaching methods included lecture, discussion, demonstration, practice, small and large group discussions and application of material to case and workflow examples. The assessment methods were congruent with the content and teaching methods including knowledge tests, psychomotor skill competency, application of the workflow process and application of concepts to case examples in the village context. Figure 1 outlines the CHW workflow process of the integration of BP monitoring with rehabilitation care. The training program was two days. Lecture, demonstration, practice and discussion were part of both days. On the second day, the focus was on workflow processes and clinical reasoning with application to case scenarios in the village context. Group 1 attended the training on the first two days, and Group 2 on the second two days of the training week. Two groups were trained separately due to limitations in the size of the training space and the need for small group discussions. Group 1 and Group 2 training was delivered by the same training team. Table 2 summarizes the training program details, teaching methods and assessment methods.

**Table 2.** Training program details.

Training Day	Teaching Content	Teaching Method	Assessment Method
Day 1	Knowledge: Blood pressure (BP) basics, rehabilitation skills rationale, CHW roles and responsibilities	Lecture, small group discussion	Written, paper-and-pencil Pre-Test
	Psychomotor Skills: BP, rehabilitation skills	Demonstration and practice, large and small group discussion	Skill Competency Observation
Day 2	Knowledge, continued: Blood pressure (BP) basics, rehabilitation skills rationale, CHW roles and responsibilities	Lecture, small group discussion	Written, paper-and-pencil Post-Test
	Psychomotor Skills, continued: BP, rehabilitation skills	Demonstration and practice, large and small group discussion	Skill Competency Observation
	Applied Understanding: Case and Workflow	Application to cases and workflow, large and small group discussion	Case examples, Workflow examples

### 2.5. Data collection

Data collected included assessment of the CHW's cognitive, psychomotor and clinical reasoning skills. Knowledge was assessed with a paper-and-pencil written multiple choice pre- and post-test on BP monitoring procedures and the integrated care workflow. The pre-test was administered after introductions on the first day and the post-test was administered after the final training activities on the second (final) day. The knowledge test was scored using an answer key by the instructors. Assessment of competent skills and reasoning included: proper use of the BP monitor, identification of important numbers on the BP monitor (systolic and diastolic BP), identification of referral criteria for known hypertension, identification of referral criteria for chronic cardiac failure, accurate recording in the Health Passport, accurate recording in the CHW Registry, accurate completion of the Referral Form, demonstration of rehabilitation skills using a reference manual (teaching patient and caregiver), demonstration of teaching stroke prevention, and application of the workflow to actual patient cases. Assessors were trained by the HBPC coordinator and physical therapist project lead to evaluate the expected skill competencies. Each skill was directly observed by an instructor and evaluated for competency. Participants were asked about potential facilitators and barriers to their work as part of the post training survey. Appendix 1 contains the training materials including the pre-post-test, skill competency list and post-training survey.

### 2.6. IRB Approval

The study was submitted to the Office of Research and Sponsored Program Institutional Review Board. Upon review of our protocol, it was approved as exempt from a need for full review under 45CFR 46.101(b). The data were coded in such a manner that the participants cannot be identified, directly or through linked identifiers. The investigators are the only persons with access to the data. All data were coded, and individual identifiers excluded from the dataset. A codebook was kept in a locked location. The individuals were not identifiable in any reporting of the findings of this study.

### 2.7. Data analyses

All results were de-identified. Descriptive frequencies (percentages) were used to describe skill competency and quantitative responses from the post-training programme survey. Given that the data were not normally distributed, the Wilcoxon Signed Rank Test (*a* = 0.05) was used to compare the means and determine if there was a significant difference in the paired outcome (pre-and post-knowledge test scores). Data analyses were undertaken using SPSS software, Version 25 [11]. This study sample size of n = 59 exceeds the sample size of 34 to achieve a power 80% at a level of significance of 5% for detecting an effect size of 0.5 [12]. Narrative responses in the post-training programme survey were analysed qualitatively using a thematic analysis. Thematic analysis included coding each participant's response, comparing responses across participants and identifying core categories (themes) [13].

## 3. Results

### 3.1. Attendance

Sixty-one CHWs were invited to attend the training and 96.7% completed both days of training (n = 59). Two participants attended only one day of training (child illness and a village funeral were the reasons for missing the second day). There was no significant difference in age or gender distribution between Groups 1 and 2. Group 2 had more participants from Traditional Authority (TA) Kalolo than Group 1 (overall there were more participants from TA Kalolo than TA Malware).

### 3.2. Knowledge pre-and post-test

The average score on the knowledge pre-test was 6.59 out of 10 (range of 2–9) and the average score on the knowledge post-test was 8.24 out of 10 (range of 3–10). There was a statistically significant improvement from the pre-posttest for Group 1, Group 2 and for both Groups combined (Table 3).

**Table 3.** Pre- and post-training knowledge for 2019 training.

Training Year	Group	N	Pre-test Mean^a^ (Standard Error, SD)	Pre-test Range	Post-test Mean^a^ (Standard Error, SD)	Post-Test Range	Z-statistic (p value)
2019	1	28	7.36 (0.225, 1.193)	5–9	8.61 (0.220, 1.166)	7–10	−3.736 (p < 0.001)*
2019	2	31	5.90 (0.319, 1.777)	2–9	7.90 (0.351, 1.955)	3–10	−4.181 (p < 0.001)*
2019	1 and 2	59	6.59 (0.219, 1.683)	2–9	8.24 (0.215, 1.654)	3–10	−5.569 (p < 0.001)*

Note: *significant at p < 0.05; ^a^ Possible score ranged from 0 to 10 with 10 being a perfect score.

### 3.3. Skills competency

One hundred percent of the participants who attended both days of training (n = 59) demonstrated all of the 10 skills at the expected level of competency (Appendix 1).

### 3.4. Application

On the final day of the training, the participants applied the workflow to patient scenarios based on real patients in the village and presented their cases. Table 4 is a summary of the cases presented.

**Table 4.** Application of training: case examples.

Patient Case	BP/Referral Decision	Rehabilitation Skills Recommended
Age: 75; Married; Chronic Cardiac Failure; 6 children. He is failing to walk because of swollen legs and difficulties in breathing.	85/55, Referred	Hand-held and cane assistance for walking
Age: 43; Hypertension. He has weakness on the left side (2 years ago). Leg and arm are weak but also, he feels pain in his head and back.	123/108, Referred	Elbow, hip and knee range of motion
Male; Age: 55; Hypertension; 3 children; Farmer; Left arm and leg are weak.	183/105, not referred. This was an incorrect decision and was corrected during the training.	Shoulder, elbow, hand and hip range of motion
Female; Age: 50; Hypertension; 5 children; Farmer; Stroke: Left arm and leg. Wants to walk alone.	99/60, not referred	Walking assistance with a cane, standing exercises
Age: 55; Chronic Cardiac Failure; Married; Farmer; Weakness of the leg and arm.	80/70, referred	Shoulder, elbow, hand, hip, knee and ankle range of motion
Age: 77; Hypertension; Married; Had a stroke 26/6/19 and his left arm and leg were weak, but he cannot talk. He has swelling and painful legs.	114/69, not referred	Shoulder, hand, hip, knee and ankle range of motion
Age: 68; Chronic Cardiac Failure; Married; 4 children; Farmer.	90/60, referred	Position changes to prevent pressure injury, feeding positioning to prevent aspiration, arm and leg range of motion
Age: 63; Hypertension; Married; 5 children; Farmer. Left leg and arm are weak. He can walk around outside with help. He gets short of breath and rests. Patient wants to walk and use his arm.	127/91, not referred	Shoulder, elbow, hand, hip, knee and ankle range of motion. Sit to stand exercise
Age: 45; Hypertension; Married; 3 children. Has left arm and leg weakness.	82/50, not referred	Shoulder range of motion, sit to stand exercise, standing exercises
Female; Hypertension; Age: 73; 4 children; Farmer, Church member. Stroke in 2017. Right arm and left are weak, can sit in chair but cannot walk around outside.	166/91, not referred	Arm and leg range of motion, sit to stand exercise, standing exercises
Age: 65; Hypertension; 3 Children; Our patient had a stroke three months ago. The left arm and leg are weak. Fingers, elbow and ankle are stiff.	180/170, referred	Shoulder, elbow, hand and hip range of motion

### 3.5. Post training survey

The post training survey included questions about the helpfulness of the training related to skill review, BP monitoring, referral criteria and documentation procedures. The survey also captured open-ended question responses about facilitators and barriers to their work. Overall, the participants reported that the training provided a helpful skill review, as well as instruction in BP monitoring, referral and documentation. Table 5 is a summary of the Likert scale responses as well as the themes in their narrative responses.

**Table 5.** Post-training survey summary.

Quantitative Results (n = 59)				
Question	A lot (%)	Some (%)	A little (%)	None (%)
Did this training help you review your skills?	68	29	2	0
Did this training help you know how to read blood pressure?	66	30	2	0
Did this training help you know when to refer?	57	36	3	0
Did this training help you know when to write in the notebook, health passport and referral form?	70	29	2	0

Qualitative Results		
Question	Themes	Exemplary Quote
What did you learn in this training? List 3 most important things you learned.	BP monitoring protocol: procedures, referral and documentation Rehabilitation Skills Communication with patients and the hospital	*“We have learned to know which patients are supposed to be referred”* *“We have also learned how to help our patients move/walk”* *“We have learned to ask out patients what they want us to do for them”*
What more would you like to learn about in the next training for physio?	More about BP including medications and management More about rehabilitation skills More about patient difficulties: oral motor, nonverbal, visually impaired, deaf More about other conditions: sickle cell, malaria, cancer, swelling	*“We should have more BP trainings on how we can do better in measuring”* *“I want to learn more skills”*
What did you learn from the follow up home visits?	Building guardian and patient relationships Seeking guidance from HPBC [Home Based Palliative Care] coordinator Learned how to use rehabilitation skills Collaborating with other volunteers	*“We learned to show our love to our patients and making good relationships with people from the villages”* *“Writing a referral to the hospital and sending text to Alex”* *“We have learned other skills from our fellow volunteers, and they call us community doctors”* *“We should move together, not alone when seeing patients”*
Describe one example of one patient's progress	Able to walk longer distances/use assistive device Able to sit up Able to move alone Able to do simple things alone Compliant to medication	*“Patient is now doing fine, is uses a cane to walk”*
Describe one example of one patient's continued difficulty	Not improving due to: Lack of medicine compliance Continues to smoke, drink Not compliant to rehabilitation program Not cooperative Lack of guardian involvement Other medical co-morbidities	*“The guardians are not taking part of the care team”*
Describe one thing that makes your work easier	Mutual respect, open communication between volunteers Training and education Encouraged by patient improvement Trainings Materials (phone, BP machine, manual/book, supplies) Teamwork and encouragement from the hospital Good relationships with chiefs and village headmen	*“I am dedicated by observing time to see my patients. I spare my own time to see these patients”* *“We should have more frequent trainings”*
Describe one thing that makes your work difficult	Transportation (long distances, lack of bicycle)—noted in 76% of responses Lack of teamwork Lack of supplies (raincoat, BP machines, phone)	*“I travel long distances to see patients, which makes work difficult”* *“Not having teamwork spirit”*

## 4. Discussion

This study demonstrated the effectiveness of a training program for CHWs which integrated BP monitoring with rehabilitation care for people with NCDs. Cognitive, psychomotor and clinical reasoning aspects of their learning were evaluated. The participants acquired knowledge (an average improvement of 1.2 points on a 10-point test), demonstrated competency with skills (100% of participants demonstrated all skills) and applied what they learned to patient cases and workflow processes (100% of participants demonstrated application of training material to cases and workflow). Given the importance of addressing NCDs in Malawi, the present study is an example of how CHWs can effectively perform rehabilitation skills, teach these skills to patients and caregivers, deliver stroke prevention education and monitor BP [1],[9]. By evaluating training effectiveness, leaders of global health initiatives can be accountable for the feasibility and sustainability of CHW education [14]. Through their presentations of clinical cases, the CHWs demonstrated direct applications of what they learned in the trainings to patient care in their villages. Their retention of knowledge and application to clinical practice serve as strong indicators of the feasibility and sustainability of the CHW training program. All of the CHWs in this study showed potential to increase access to integrated care in this low-resource setting. Our findings provide evidence for Perry et al.'s (2012) description of CHWs as “the world's most promising health workforce resource for enabling health systems in resource-constrained settings to reduce the burden of disease” [15].

In the post-training survey, CHWs reported that they will be able to apply their new skills and knowledge to improve the health of the villages they serve. Similar to CHWs in other settings, participants reported the lack of transportation, teamwork, and resources as barriers to their success [16]–[18]. Participants also noted facilitators of their success, including trainings and opportunities to collaborate—also reported by Greenspan et al. (2013) [19].

The WHO initiative for UHC emphasizes integrated, person-centered care with a focus on optimal processes of care delivery and patient experience [4]. Studies have shown that CHWs are cost-effective and integral to the success of health systems in Sub-Saharan Africa [6],[20]. Prior to this training, the CHWs showed they were successful in reaching people with disabilities with rehabilitation care. [21],[22]. With BP monitoring, their scope of care was expanded. They now have a workflow process for monitoring BP, providing preventative education to patients and families and connecting them to the local hospital. In this setting, CHWs improve the patient experience by combining preventative and rehabilitation services into home-visits and by facilitating connections between the community and local access points of health care such as the hospital. The quality of the patient experience is potentially improved because of the integrated, person-centered approach.

A recent systematic review which included 127 studies from low- and middle-income countries highlighted that people with disabilities have higher healthcare needs yet encounter more barriers to accessing services [23]. This lack of health coverage and access not only violates the rights of people with disabilities under international law but also results in poorer health outcomes [23]. The WHO recommends integrating CHWs into health systems, as they can improve health equity by improving access to people who may not have the means for transport to a hospital [24]. Because they are embedded into the communities in which they serve, CHWs can identify needs in their communities and provide a healthcare access point. A study undertaken in India demonstrated that CHW training in management of disabilities led to earlier identification of people with disabilities in rural settings [25]. In Malawi, accessing health care is difficult and health inequities are widespread, but the presence of CHWs may alleviate some of these burdens. The HBPC program at a rural hospital in Malawi demonstrated that training CHWs in basic rehabilitation and monitoring skills is effective over a seven-year period. The CHWs applied their knowledge to ongoing care in the villages and they reported positive changes in health outcomes. Our findings help demonstrate that training CHWs can be an effective way to help bridge the gap in health care access for people with disabilities in resource-limited countries.

## 5. Conclusion

This is the first study to demonstrate the effectiveness of incorporating preventative care in a community-based rehabilitation program. Integrated care improves the quality of the patient experience and access to medical services in underserved areas. Global health initiatives in these low-resource settings must focus on creating sustainability by cultivating relationships with local health providers [26]. CHWs play a vital role in these healthcare systems by extending the access point for care and delivering care with a person-centered focus. Future studies will be undertaken to identify the barriers in the present CHW model that prevent patients from receiving appropriate medical care. The national NCD report in Malawi highlights the importance of understanding structural determinants of health in relation to NCD management [1]. We will continue to investigate how these factors affect the management of NCDs in this setting but see potential for this ongoing care model to have an impact on health access for people with chronic conditions.
