# Supplementary material for: Integrated care by community health workers in Malawi: Rehabilitation and blood pressure monitoring
Source: AIMS Public Health. 2021 Jan 28;8(1):110–23. doi: 10.3934/publichealth.2021009 (PMC7870379; doi:10.3934/publichealth.2021009)
Supplement: Supplementary file 1 [file publichealth-08-01-009-s001.pdf]

AIMS Public Health

AIMS Public Health, 8(1): 110–123.
DOI: 10.3934/publichealth.2021009
Received: 25 November 2020
Accepted: 26 January 2021
Published: 28 January 2021

http://www.aimspress.com/journal/aimsph

*Research article*

# Integrated care by community health workers in Malawi: Rehabilitation and blood pressure monitoring

**Athenie Galvez[1,*], Jordan Waite[2], Kyle Jureidini[3] and Kathryn C Nesbit[4]**

[1] Presidio Sport & Medicine, San Francisco, CA, USA
[2] Orthopedic and Neurological Rehabilitation, Los Gatos, CA, USA
[3] Body Mechanix Physical Therapy, Simi Valley, CA, USA
[4] UCSF/SFSU Graduate Program in Physical Therapy, San Francisco, CA, USA

* **Correspondence:** Email: atheniegalvez@gmail.com; Tel: 510-304-2014.

## Appendix 1. Training Materials

Pre Test/Post Test (10 multiple choice questions, one point per question, 0–10 points possible)

**Knowledge questions (Pre- and Post-Test):**

1. What is blood pressure?
   A. The messages from the brain
   B. The pressure of the blood in the vessels
   C. The beat of the heart
2. At what level should the blood pressure machine be placed?
   A. Heart level
   B. Eye level
   C. Stomach level
3. What should you do when you are taking the patient's blood pressure?
   A. Provide stroke prevention education

B. Do skill #5
C. Text Alex

4. When should you refer a patient with HTN?
   A. When the bottom number is more than 100
   B. When the bottom number is less than 100
   C. When the top number is less than 100
5. When should you refer a patient with CCF?
   A. When the bottom number is more than 100
   B. When the top number is less than 100
   C. When the top number is more than 100
6. Where should you document the blood pressure?
   A. In the notebook, health passport and referral form (if referred)
   B. In the health passport only
   C. On the referral form only
7. When texting Alex with a question about exercises what should you include?
   A. The patient's age and height
   B. The patient's goals
   C. The patient's caregiver name
8. After you know the patient has HTN or CCF, what should you do next?
   A. Collect your supplies: Blood pressure Machine, notebook, referral form, health passport
   B. Text Alex
   C. Teach physiotherapy skills
9. If a patient needs a referral, what should you do?
   A. Complete the referral form and text Alex
   B. Teach physiotherapy skills
   C. Nothing
10. If you have a new patient, after you take the blood pressure, what should you ask?
   A. What is the patient's age
   B. What the patient wants to do (patient goals)
   C. Where is the patient's toilet

**Skill competency list**

1. Proper use of BP monitor
2. Identification of important numbers on BP monitor
3. Identification of referral criteria for known HTN on BP monitor
4. Identification of referral criteria for CCF on BP monitor
5. Proper recording in Health Passport
6. Proper recording in Notebook
7. Proper completion of Referral Form
8. Demonstrates rehabilitation skills using manual (teaching patient and caregiver)

9. Demonstrating teaching stroke prevention
10. Application to patient case: BP monitoring, teaching skills and communicating via SMS to patient

**Post-Training Survey**

Name Village

1. What did you learn in this training? List 3 most important things you learned.
   1.
   2.
   3.
2. Did this training help you review your skills?
   No A little Some A lot
3. Did this training help you know how to read blood pressure?
   No A little Some A lot
4. Did this training help you know when to refer?
   No A little Some A lot
5. Did this training help you know when to write in the notebook, health passport and referral form?
   No A little Some A lot
6. What more would you like to learn about in the next training for physio?
7. What did you learn from the follow up home visits?
8. Describe one example of one patient's progress.
9. Describe one example of one patient's continued difficulty.
10. Describe one thing that makes your work easier.
11. Describe one thing that makes your work difficult.

AIMS Press © 2021 the Author(s), licensee AIMS Press. This is an open access article distributed under the terms of the Creative Commons Attribution License (http://creativecommons.org/licenses/by/4.0)
